# Multilayer modulation of the proteasome: new strategies for neuroprotection

**DOI:** 10.3389/fnmol.2026.1748434

**Published:** 2026-01-26

**Authors:** Maxim Sokolov, Hiroaki Taniguchi, Jonasz Jeremiasz Weber

**Affiliations:** 1 Departments of Ophthalmology, Biochemistry and Molecular Medicine, Neuroscience, West Virginia University, Morgantown, WV, United States; 2 Department of Experimental Embryology, Institute of Genetics and Animal Biotechnology of the Polish Academy of Sciences, Jastrzȩbiec, Poland; 3 African Genome Center, University Mohammed VI Polytechnic (UM6P), Hay Moulay Rachid, Ben Guerir, Morocco; 4 Department of Human Genetics, Ruhr University Bochum, Bochum, Germany; 5 Institute of Medical Genetics and Applied Genomics, Eberhard Karls University Tübingen, Tübingen, Germany

**Keywords:** 20S proteasome, neurodegeneration, neuronal proteostasis, Nrf1 (NFE2L1), proteaphagy, proteasome regulation, transcriptional control, xenogeneic proteostasis

## Introduction

Neurodegenerative diseases—including Alzheimer's, Parkinson's, and Huntington's disease, as well as amyotrophic lateral sclerosis (ALS)—share a defining pathological feature: the accumulation of misfolded, aggregation-prone proteins that overwhelm neuronal proteostasis networks. The consequent failure to efficiently clear not only disease-associated proteins but also other misfolded or damaged substrates leads to toxic buildup, contributing to synaptic dysfunction, apoptotic signaling, and ultimately widespread neuronal loss (Ciechanover and Kwon, 2015; Hipp et al., 2019). Under physiological conditions, neurons rely predominantly on the ubiquitin–proteasome system (UPS) for selective protein degradation and maintenance of proteome integrity (Schmidt et al., 2021). Sustaining proteostasis—the delicate balance between protein synthesis, folding, and degradation—is therefore critical for neuronal survival throughout life.

Multiple layers of proteasomal regulation have been described, representing both potential vulnerabilities and therapeutic entry points in neurodegenerative disease. These include transcriptional control of proteasome subunit expression, assembly and activity modulation through post-translational modifications, substrate recognition dynamics, and cross-talk with other proteolytic machineries (Rousseau and Bertolotti, 2018). However, many of these processes remain only partially understood or have not yet been explored in the neuronal context. In this *Opinion* article, we highlight four complementary forms of proteasomal regulation and functional augmentation—ranging from transcriptional control, through stimulation of 20S core particles and proteolytic modulation, to xenogeneic enhancement. We propose that a deeper dissection of these mechanisms and their translation into the framework of neurodegenerative pathology could open new avenues for therapeutic intervention.

We begin by examining how the transcriptional landscape governs proteasome biogenesis and activity, laying the foundation for understanding how cells sense and adapt to proteotoxic stress.

## Transcriptional regulation of proteasome homeostasis: transcription factors as master regulators

The transcriptional control of proteasome biogenesis represents a critical layer of regulation, coordinated by several stress-responsive transcription factors (Kamber Kaya and Radhakrishnan, 2021; Motosugi and Murata, 2019). Among these, Nrf1 (NFE2L1) functions as a master regulator of proteasome homeostasis. Nrf1 is a basic leucine zipper (bZIP) transcription factor that activates proteasome subunit genes in response to proteotoxic stress. Hossein et al. summarized Nrf1 as a central regulator of proteostasis in neurodegenerative diseases, highlighting its interplay with autophagy, ferroptosis, and the proteasome (Khodadadi et al., 2025). Unlike Nrf2 (NFE2L2), which primarily regulates antioxidant defense, Nrf1 serves as a “guardian” of proteasome integrity—sensing proteasomal inhibition and restoring protein-degradation capacity (Łuczyńska et al., 2024).

Studies using Nrf1-deficient models have revealed that impaired induction of proteasome genes triggers neurodegenerative phenotypes (Lee et al., 2011). Activation of Nrf1 requires proteolytic cleavage and release from the endoplasmic reticulum (ER) membrane, followed by nuclear translocation (Chavarria et al., 2023). Additional regulatory layers likely involve transcriptional partners, post-translational modifications, and feedback signals from the broader proteostasis network.

Beyond Nrf1, several other transcription factors contribute to proteasome regulation. Nrf2 can also induce certain proteasome subunit genes (Pajares et al., 2017). Moreover, transcription factors including PAX4 and FOXO3 (Gilda et al., 2024) participate in regulating proteasome-related genes. Despite their collective importance, our understanding of these transcriptional networks remains limited. The molecular mechanisms governing their interplay—and the influence of epigenetic modifiers that shape chromatin accessibility—are not yet fully defined. Further studies are needed to elucidate how these factors integrate to maintain neuronal proteostasis and resilience under neurodegenerative stress.

While transcriptional programs govern proteasome abundance and long-term capacity, neurons also deploy faster, post-transcriptional mechanisms to adapt proteolytic activity to acute proteotoxic stress. One such mechanism is the mobilization and stimulation of the free 20S proteasome core, which enables rapid, ubiquitin-independent degradation of damaged and intrinsically disordered proteins.

## Mobilization and stimulation of the 20S proteasome: adaptation of proteolytic capacity

The proteasome exists in multiple functional assemblies, with the 26S proteasome mediating ubiquitin- and ATP-dependent degradation of structured proteins, and the free 20S core particle carrying out ubiquitin-independent proteolysis of intrinsically disordered and oxidatively damaged proteins (Kisselev et al., 2002; Raynes et al., 2016). Multiple studies have demonstrated that the 20S proteasome is not a latent protease but rather constitutes a highly abundant and catalytically competent pool capable of degrading intrinsically disordered proteins (IDPs) without assistance from regulatory particles (Alvarez-Castelao et al., 2014; Raynes et al., 2016). This property is particularly relevant in aging and neurodegenerative disease, where the accumulation of disordered, and aggregation-prone proteins places increased demands on ubiquitin-independent degradation pathways.

Several pharmacological strategies have been identified that stimulate 20S proteasome activity. Early studies identified small molecules such as betulinic acid that enhance chymotrypsin-like activity of the 20S proteasome, although these compounds often exhibit limited activity under physiological conditions (Huang et al., 2007). More recent work has identified bona fide small-molecule stimulators that promote gate opening of the 20S core and selectively enhance degradation of IDPs such as α-synuclein and tau, while sparing structured proteins (Fiolek et al., 2021a,b; Jones et al., 2017). These compounds act independently of ubiquitination and do not require association with the 19S regulatory particle, supporting the concept that targeted stimulation of the 20S proteasome can selectively enhance clearance of proteotoxic substrates.

Notably, N-methyl-D-aspartate receptor (NMDAR) antagonists, including memantine and ketamine, were recently shown to robustly enhance 20S proteasome activity in cells and *in vivo*, leading to selective depletion of intrinsically disordered and aggregation-prone proteins such as tau, amyloid precursor protein, and α-synuclein (Sahin et al., 2025). These findings provide a clinically relevant example of pharmacological 20S stimulation and suggest that enhancement of ubiquitin-independent proteolysis may contribute to the therapeutic efficacy of NMDAR antagonists in neurodegenerative and neuropsychiatric disorders.

Peptide- and peptidomimetic-based activators provide further mechanistic insight into 20S stimulation. Short peptides containing hydrophobic or basic motifs, including HbYX-containing peptides and Tat-derived peptides, bind to intersubunit pockets on the α-ring and allosterically promote gate opening (Chuah et al., 2023; Kisselev et al., 2002; Osmulski et al., 2020). Structural and biochemical analyses indicate that these interactions destabilize the closed conformation of the 20S gate, thereby increasing substrate access to the catalytic chamber (Cekała et al., 2022; Osmulski et al., 2020). Importantly, cyclic peptide proteasome stimulators and other stabilized peptide scaffolds have been shown to enhance degradation of highly disordered proteins both *in vitro* and in cell-based assays, while leaving folded proteins largely unaffected (Nelson et al., 2024). Together, these studies establish that allosteric engagement of the α-ring represents a generalizable mechanism for selectively stimulating ubiquitin-independent proteolysis.

In addition to pharmacological approaches, genetic strategies that directly destabilize the 20S gate provide definitive evidence for the biological consequences of 20S stimulation. Deletion of N-terminal gating residues in α subunits generates constitutively open-gate proteasomes with markedly increased 20S activity (Anderson et al., 2022). *In vivo* studies using an α3ΔN open-gate mutant demonstrate enhanced degradation of intrinsically disordered and aggregation-prone proteins, reduced oxidative damage, improved endoplasmic reticulum–associated degradation (ERAD) and increased organismal stress resistance and lifespan (Salcedo-Tacuma et al., 2025). These effects occur independently of canonical unfolded protein response signaling, supporting the existence of a distinct “20S pathway” of proteostasis that directly mitigates proteotoxic stress.

Collectively, these findings demonstrate that stimulation of the 20S proteasome—through small molecules, peptides, or genetic gate opening—selectively enhances degradation of intrinsically disordered and misfolded proteins. This mode of proteasome activation represents a mechanistically distinct and potentially therapeutically relevant strategy for alleviating proteostasis defects associated with aging and neurodegenerative disease (Jones et al., 2017; Sahin et al., 2025).

Beyond direct stimulation of the proteasomal core through small molecules, peptides, or genetic strategies, an additional layer of regulation operates post-translationally, where proteolytic mechanisms fine-tune proteasome abundance, stability, and activity in real time.

## Post-translational modulation of proteasome function: proteolytic mechanisms in control

Proteasomal activity is regulated not only by complex upstream transcriptional programs and interactions with a wide range of cofactors but also by post-translational modifications, which provide a dynamic and versatile layer of control (Arkinson et al., 2025). Among glycosylation, acetylation, and ubiquitination, phosphorylation stands out, with nearly 300 modification sites identified across proteasomal subunits—underscoring the proteasome's remarkable regulatory complexity (Türker et al., 2021). Yet, despite its role as a proteolytic machinery, surprisingly little is known about how proteolytic effectors modulate proteasomal function.

One particularly intriguing example of this regulation is proteaphagy—the selective autophagic clearance of excess or inactive proteasomes. Initially characterized in *Arabidopsis* and yeast, this process is mediated by interactions between the proteasomal ubiquitin receptor Rpn10 and ATG8, or between Cue5 and the chaperone Hsp42, respectively (Marshall et al., 2015, 2016). The discovery that several factors specifically involved in proteaphagy are dispensable for general autophagy highlights a dedicated and potentially tunable mechanism for proteasome quality control (Waite et al., 2022). Such selectivity may offer an appealing opportunity for targeted manipulation of proteostasis in neurons, where proteasomal turnover is crucial for maintaining cellular health.

Notably, the E3 ubiquitin ligase STUB1/CHIP—implicated in multiple neuropathological contexts and whose mutations cause several neurodegenerative syndromes (Zhang et al., 2020)—has been shown to direct proteasomes toward aggresomal sequestration and subsequent autophagic degradation in mammalian cells (Choi et al., 2020). This observation suggests that proteaphagy may constitute an underappreciated route for regulating proteasomal homeostasis in the nervous system.

While proteaphagy removes entire proteasomes from the system, other proteolytic events have been shown to modify proteasomal activity and integrity at the subunit level. Caspases, primarily associated with programmed cell death (Dho et al., 2025), can cleave proteasomal subunits. Caspase cleavage of proteasomal proteins is generally associated with proteasomal inactivation during apoptosis, where subunits of both the 19S regulatory complex and the 20S core are cleaved, leading to stabilization of pro-apoptotic factors (Adrain et al., 2004; Gray et al., 2010; Jang et al., 2007; Sun et al., 2004).

In skeletal muscle, caspase-3 has been observed to cleave Rpt2, Rpt5, and Rpt6, with proteolysis of Rpt5 in myoblasts decreasing proteasomal activity, while cleavage of Rpt2 and Rpt6 in myotubes increases activity—potentially contributing to muscle-wasting conditions (Wang et al., 2010). This complexity of downstream effects suggests that caspase cleavage not only disrupts proteasome function but may also activate it, revealing another layer of regulatory control.

Likewise, calcium-activated calpains—considered protein function-modulating proteases with various associations to neurodegenerative disorders (Incebacak Eltemur et al., 2022; Ono et al., 2016)—have been shown to negatively regulate proteasomal stability by cleaving Rpn10 in neurons, triggered by mitochondrial dysfunction and contributing to disrupted proteostasis. These findings, however, only begin to uncover what may be a far more intricate network of proteolytic mechanisms influencing proteasome dynamics.

Despite the complexity and adaptability of these endogenous regulatory systems, their capacity to counteract aggregation-prone proteins appears limited in neurodegenerative settings. This limitation has inspired exploration beyond mammalian biology—to identify and harness proteostasis solutions that evolved in other kingdoms of life.

## Cross-kingdom proteostasis: lessons from microbial systems

Despite the presence of sophisticated molecular chaperones and proteasomes, cytotoxic species of proteins involved in neurodegenerative disorders resist clearance, leading to progressive neuronal dysfunction and death. This therapeutic impasse invites exploration of alternative proteostasis mechanisms that have evolved outside the animal kingdom.

Unicellular organisms—including protozoa, bacteria, and archaea—thrive in extreme or fluctuating environments that promote protein denaturation and aggregation. To survive, these organisms have evolved robust protein quality control systems capable of maintaining proteostasis under stress conditions lethal to mammalian cells. Because the fundamental principles of protein folding are conserved across all domains of life, xenogeneic chaperones and proteasomes from such organisms may recognize and degrade misfolded proteins that evade mammalian degradation pathways.

Among the best-characterized examples is the yeast disaggregase Hsp104, a hexameric AAA+ ATPase absent from metazoans. Expression of Hsp104 in animal models reduces proteotoxic aggregates and ameliorates disease phenotypes in Huntington's (Vacher et al., 2005), Alzheimer's, and Parkinson's (Lo Bianco et al., 2008; Vashist et al., 2010) disease models. Notably, Hsp104 can be *tuned* for enhanced or selective activity by mutating specific residues (Jackrel et al., 2014), demonstrating its engineering potential as a customizable proteostasis factor. Similarly, the prokaryotic Hsp104 homolog ClpG suppresses the toxicity of TDP-43, FUS, and α-synuclein—proteins implicated in ALS, frontotemporal dementia, and Parkinson's disease—when expressed in yeast (March et al., 2020). These findings illustrate that microbial disaggregases can counteract diverse aggregation-prone proteins associated with human neurodegeneration.

Complementary results have emerged from studies of archaeal proteasomes. Expression of the archaeal 20S proteasome from *Methanosarcina mazei* in mammalian cells increased degradation of aggregation-prone proteins, including mutant superoxide dismutase-1 (ALS), mutant androgen receptor (spinal and bulbar muscular atrophy), tau (Alzheimer's disease), and α-synuclein (Parkinson's disease) (Yamada et al., 2006). Moreover, the *gateless* 20S proteasome from *Thermoplasma acidophilum* was able to digest polyglutamine (polyQ) peptides and proteins—features characteristic of Huntington's disease and spinocerebellar ataxias—whereas eukaryotic 20S and 26S proteasomes failed to cleave within polyQ stretches (Venkatraman et al., 2004). These findings suggest that archaeal proteasomes possess unique catalytic properties that could complement or surpass the substrate specificity of their eukaryotic counterparts.

The expanding insights from microbial and archaeal systems highlight not only alternative routes to maintaining proteostasis but also the remarkable plasticity of the degradation machinery itself. These discoveries invite a broader question: how can we best integrate such evolutionary lessons with the cell's own finely tuned proteasomal networks to develop effective neuroprotective strategies?

## Discussion

Targeting proteasomal degradation as a therapeutic avenue in neurodegeneration has long been recognized and remains an area of intense investigation. Efforts have largely focused on developing proteasome activators and regulators (Church and Margolis, 2024; Njomen and Tepe, 2019) or on strategies that facilitate targeted substrate delivery to the proteasome (Gregory et al., 2024; Zhao et al., 2022). Yet, comparatively little attention has been given to the more nuanced regulatory dimensions discussed here—those that govern proteasomal function at the transcriptional, post-translational, and even cross-kingdom levels. These mechanisms likely represent only the first glimpses of a far more sophisticated regulatory landscape shaping proteasome behavior and capacity.

At the same time, neurons possess complex endogenous mechanisms to counter proteotoxic stress. Transcriptional and post-translational regulation of the proteasome constitute the central adaptive axis of neuronal proteostasis. By dynamically adjusting proteasome abundance, composition, and activity, neurons can respond to fluctuating proteotoxic demands over the course of aging and disease. Understanding how these intrinsic pathways preserve proteasomal capacity—and how they fail under chronic stress—remains essential for identifying therapeutic entry points that reinforce neuronal resilience.

Beyond transcriptional adaptation, neurons can also rapidly reconfigure proteolytic capacity through mobilization of pre-existing proteasome pools. In addition to transcriptional and post-translational regulation of the ubiquitin/ATP-dependent 26S proteasome, accumulating evidence highlights a central role for the ubiquitin- and ATP-independent 20S proteasome in neuronal proteostasis, particularly under oxidative stress. A substantial fraction of the cellular proteasome pool exists as free 20S particles, which increase with aging and disease, and the 20S proteasome is primarily responsible for the degradation of oxidatively damaged and intrinsically disordered proteins while remaining active under conditions that destabilize the 26S complex (Jung and Grune, 2008; Jung et al., 2014; Pickering and Davies, 2012). Recent studies further demonstrate that 20S proteasome activity can be pharmacologically enhanced by small molecules, including the clinically used NMDAR antagonist memantine, which increases multiple proteolytic activities of the 20S core (Sahin et al., 2025). These findings suggest that direct modulation of 20S proteasome function represents an immediately tractable therapeutic strategy that may complement approaches focused on 26S regulation (Njomen and Tepe, 2019).

At a further regulatory layer, proteasome abundance and functionality are shaped by proteolytic and post-translational events, including controlled subunit processing, stabilization or destabilization of complexes, and selective degradation of entire proteasomes via proteaphagy. Such mechanisms allow neurons to fine-tune proteasome activity spatially and temporally, but may also become maladaptive under chronic stress. Preventing excessive proteaphagy or stabilizing functional proteasome assemblies could therefore help preserve proteostatic balance without inducing cytotoxic hyperactivation.

While these endogenous mechanisms provide powerful means to adapt proteasomal capacity, they may ultimately be insufficient in the face of sustained proteotoxic stress. Emerging evidence suggests that complementing mammalian proteostasis with xenogeneic chaperones and proteasomes may offer an unconventional but promising means to enhance the clearance of aggregation-prone proteins. This “cross-kingdom proteostasis” approach harnesses billions of years of evolutionary adaptation to extreme environments, offering catalytic, or structural properties absent from mammalian systems. Before clinical translation, however, rigorous *in vivo* evaluation is required to address safety, expression control, and immunogenicity. If feasible, introducing xenogeneic proteostasis components could represent a genuine paradigm shift in the treatment of protein-misfolding disorders.

Across these regulatory scales—from transcriptional control and 20S mobilization to post-translational fine-tuning and cross-kingdom augmentation—the proteasome emerges as an active sentinel and sculptor of neuronal proteostasis. The converging evidence suggests that neuroprotection may not require a simple increase in proteasomal activity but rather a smarter modulation of *when, where*, and *how* proteasomes act. Reinforcing endogenous adaptive programs through Nrf1 activation, stabilizing proteasome complexes, or preventing maladaptive proteaphagy may preserve homeostasis while minimizing unintended cytotoxic effects.

**Figure 1:**
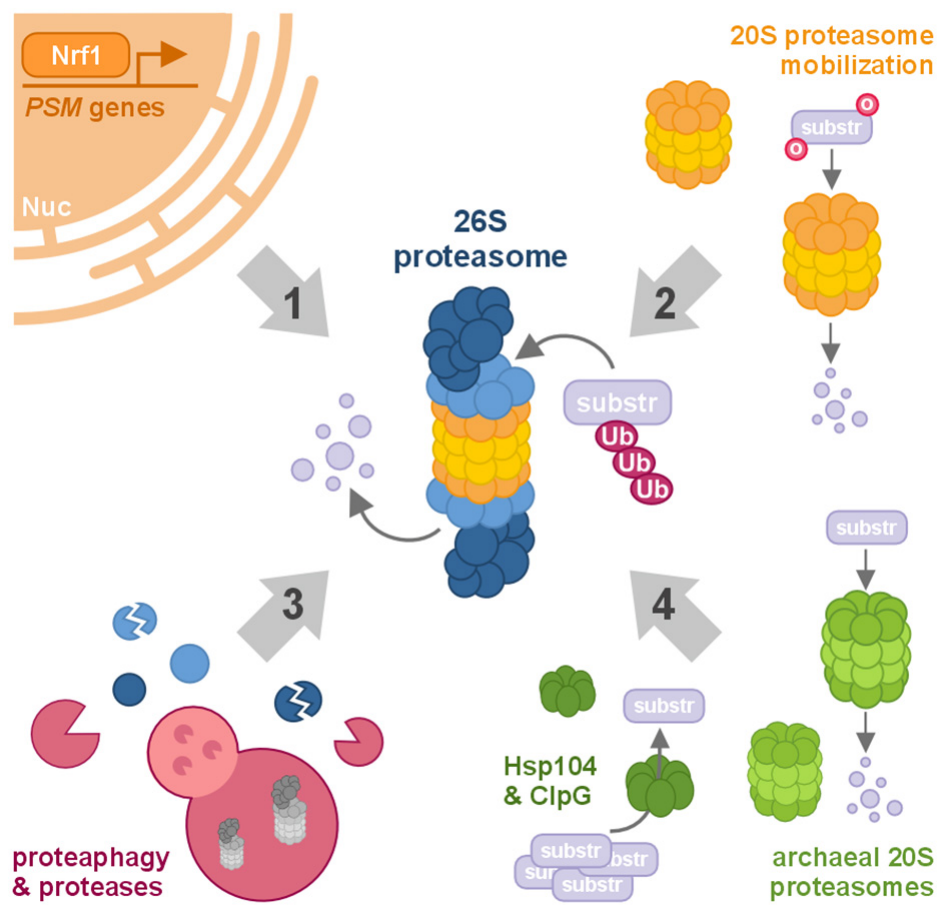
*Modulating proteasomal function*. Schematic overview of the major forms of proteasomal regulation and augmentation discussed in this *Opinion* article. Proteasomes are regulated at multiple levels that influence their abundance, substrate selectivity, and catalytic activity, and these mechanisms can be exploited to enhance the degradation of damaged, misfolded, or aggregated proteins relevant to neurodegenerative disease. **(1)** *Biogenesis:* proteasome production is controlled by the transcription of proteasomal (*PSM*) genes, regulated by factors such as Nrf1. **(2)** *20S proteasome stimulation:* the existing cytosolic pool of free 20S core particles can be activated to degrade oxidatively damaged proteins independently of ubiquitination. **(3)** *Proteolytic modulation:* proteasome abundance and activity can be altered through proteolytic mechanisms, including autophagosomal removal of entire complexes via proteaphagy or cleavage of individual proteasomal subunits by specific proteases. **(4)** *Xenogeneic augmentation:* when endogenous proteostasis is insufficient, proteasomal function may be supported by introducing xenogeneic factors such as microbial disaggregases (e.g., Hsp104, ClpG) or archaeal proteasomes. Nuc, nucleus; o, oxidative damage; substr, substrate protein targeted for degradation; Ub, ubiquitination.

Taken together, these perspectives underscore that proteostasis via the proteasome is a multilayered, evolutionarily conserved network integrating transcriptional regulation, subunit mobilization and stimulation, and proteolytic modulation, which can potentially be expanded by integrating xenogeneic augmentation in a eukaryotic context (Figure 1). Future research should aim to bridge these layers, combining the robustness of microbial systems with the precision of mammalian regulatory networks. Such an integrative framework may pave the way for the rational design of proteostasis-centered therapeutics.

In this vision, neuroprotection shifts from targeting individual toxic proteins to re-engineering the cell's proteostasis architecture itself. The proteasome—once viewed merely as the terminus of protein degradation—may instead become the central hub for sustaining neuronal identity, resilience, and longevity. Ultimately, understanding and reprogramming proteasomal regulation across molecular and evolutionary scales may redefine how we approach neurodegeneration, moving the field beyond symptomatic relief toward rebuilding the foundations of neuronal proteostasis itself.
